# Supplementary material for: A Study of Small Intestinal Epigenomic Changes Induced by Royal Jelly
Source: Cells. 2024 Aug 25;13(17):1419. doi: 10.3390/cells13171419 (PMC11393943; doi:10.3390/cells13171419)
Supplement: Supplementary file 1 [file cells-13-01419-s001.zip › Supplementary_figure1.pdf]

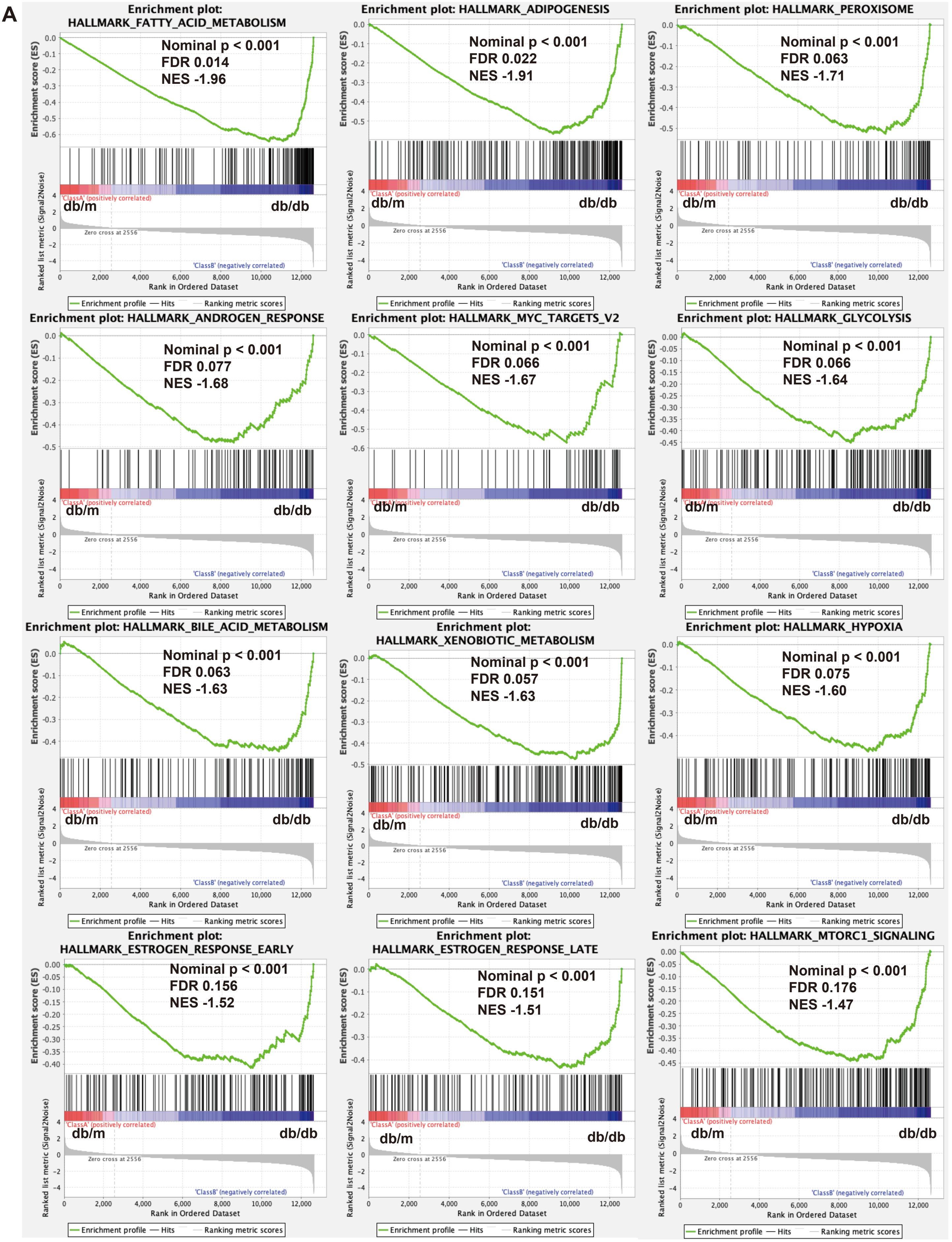

Supplementary figure 1.

A

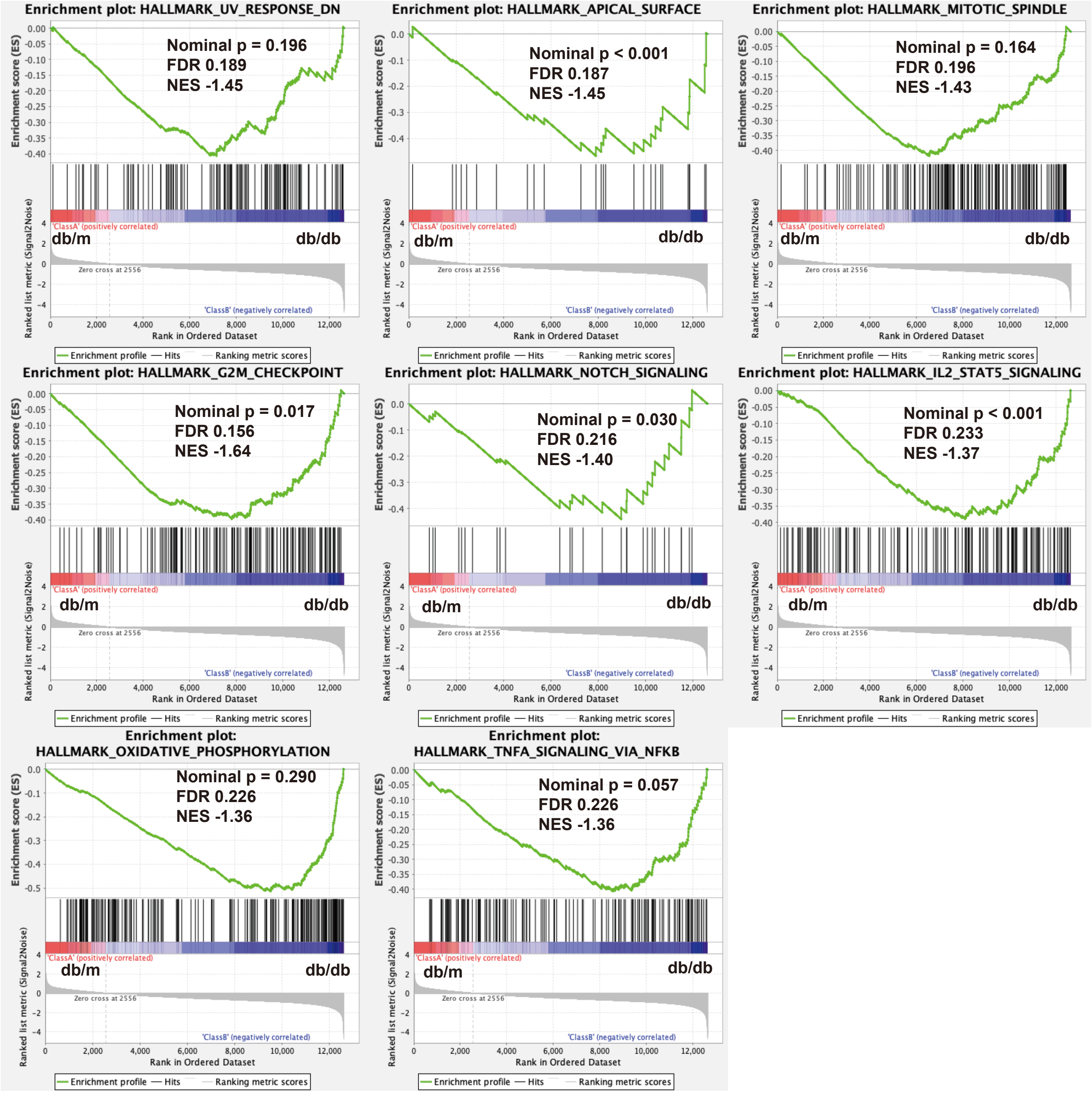

B

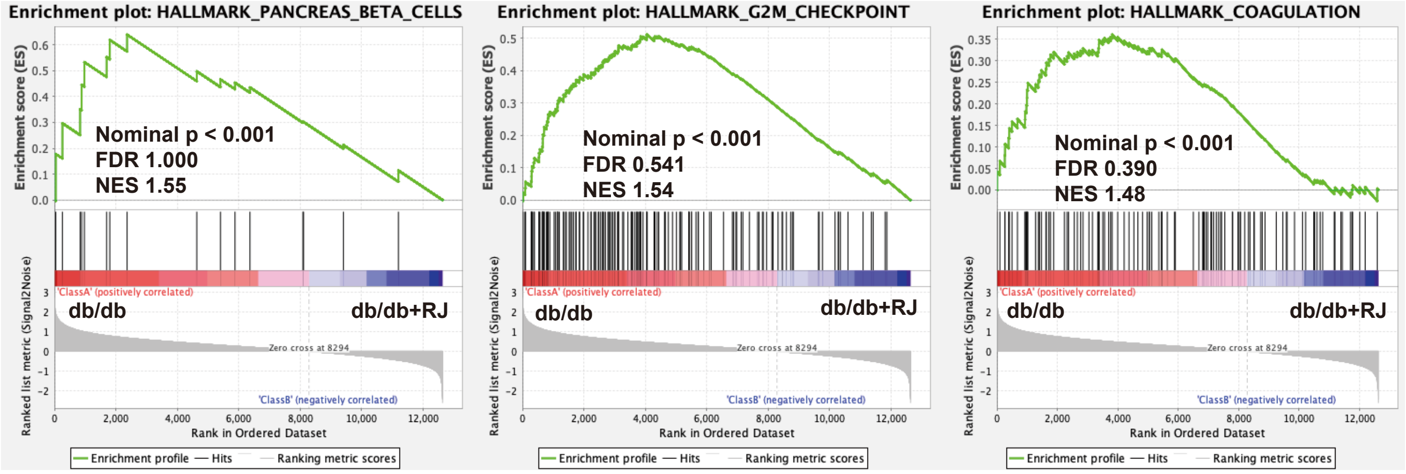

Supplementary figure 1.

C

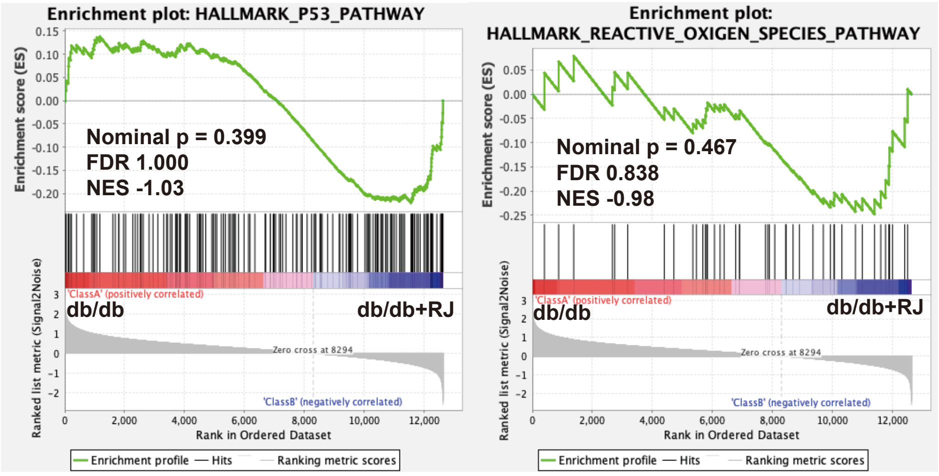

Supplementary figure 1.
The top of the figure plots the enrichment score for each gene, while the bottom of the plot shows the value of the ranking metric moving down the list of ranked genes in the small intestine. y-axis: value of ranking metric; x-axis: rank of all genes.
(A) Comparison between db/m mice and db/db mice. Gene sets were enriched in db/db mice with statistically significant differences. (B) Comparison between db/db mice and db/db+RJ mice. Gene sets were enriched in db/db mice with statistically significant differences. (C) Comparison between db/db mice and db/db+RJ mice. Gene sets were enriched in db/db+RJ mice with statistically significant differences.

Supplementary figure 1.
